# Ferroptosis resistance in cancer cells: nanoparticles for combination therapy as a solution

**DOI:** 10.3389/fphar.2024.1416382

**Published:** 2024-06-19

**Authors:** Kodzo Prosper Adzavon, Weijian Zhao, Xuesong He, Wang Sheng

**Affiliations:** College of Chemistry and Life Science, Beijing University of Technology, Beijing, China

**Keywords:** ferroptosis, ferroptosis resistance, nanoparticles, combinatory therapy, cancer therapy

## Abstract

Ferroptosis is a form of regulated cell death (RCD) characterized by iron-dependent lipid peroxidation. Ferroptosis is currently proposed as one of the most promising means of combating tumor resistance. Nevertheless, the problem of ferroptosis resistance in certain cancer cells has been identified. This review first, investigates the mechanisms of ferroptosis induction in cancer cells. Next, the problem of cancer cell resistance to ferroptosis, as well as the underlying mechanisms is discussed. Recently discovered ferroptosis-suppressing biomarkers have been described. The various types of nanoparticles that can induce ferroptosis are also discussed. Given the ability of nanoparticles to combine multiple agents, this review proposes nanoparticle-based ferroptosis cell death as a viable method of circumventing this resistance. This review suggests combining ferroptosis with other forms of cell death, such as apoptosis, cuproptosis and autophagy. It also suggests combining ferroptosis with immunotherapy.

## 1 Introduction

Cancer remains one of the world’s biggest killers, despite considerable progress in diagnostic and therapeutic techniques (Siegel et al., 2021; Sung et al., 2021). The challenges of cancer therapy are manifold. They include issues such as drug resistance, tumor heterogeneity and deleterious side effects on normal tissues (Alfarouk et al., 2015; Kudelova et al., 2022; Romani, 2022). These obstacles call for the development of innovative therapeutic strategies capable of targeting cancer cells more effectively, while circumventing the problem of resistance.

One such emerging strategy is the induction of ferroptosis, a form of regulated cell death (RCD). Ferroptosis is characterized by the accumulation of iron-dependent lipid peroxidation. Unlike other RCDs such as apoptosis and necrosis, ferroptosis targets the vulnerability of cancer cells to iron overload and oxidative stress, offering a unique angle for tackling drug-resistant and difficult-to-treat cancers (Wang Huan et al., 2023). The significance of ferroptosis in cancer treatment lies not only in its potential to eliminate cancer cells but also in its capacity to induce a form of cell death that is fundamentally different from other RCD, thereby potentially overcoming resistance mechanisms. Ferroptosis is characterized by a complex interplay of processes leading to cell death due to reactive oxygen species (ROS) attacking membrane lipids. In particular, it involves the disintegration of the cell membrane, mitochondria or various organelles (Do et al., 2023; Krusenstiern et al., 2023; Lyamzaev et al., 2023). This event is a consequence of multiple changes at the cellular level (Logie et al., 2021; Ma et al., 2022). These transformations are attributed to increased lipid peroxidation, which disrupts membrane integrity, leading to a loss of normal membrane permeability (Mortensen et al., 2023). This intensification of lipid peroxidation plays a central role in the ferroptosis process, effectively serving as a death signal for the cell (Mortensen et al., 2023; Usukhbayar et al., 2023). Notably, ferroptosis is driven by four interdependent mechanisms: iron accumulation, reactive oxygen species (ROS) generation, lipid peroxidation, and glutathione depletion. These interconnected pathways act synergistically to induce this unique form of cell death, offering a nuanced understanding of the ferroptosis process and its impact on cancer therapy (Xu et al., 2023).

Nanotechnology offers a cutting-edge platform for inducing ferroptosis in a targeted manner. Nanoparticles can be engineered to induce ferroptosis or deliver ferroptosis-inducing agents specifically to cancer cells, thereby reducing off-target effects (Cai et al., 2022; Abu-Serie, 2023; Zhang D. et al., 2023). Moreover, the physicochemical properties of nanoparticles, such as size, shape, and surface characteristics, can be precisely controlled to optimize their delivery and therapeutic efficiency (Tian et al., 2022; Wang Bingqing et al., 2023). Nanoparticles also offer the advantage of combining multiple therapeutic agents, enabling a synergistic approach to induce ferroptosis at the same time as other forms of cell death, thus widening the therapeutic window (Zhang J. et al., 2023). This review explores the versatile role of nanoparticles that trigger ferroptosis to treat cancer. These findings also suggest how these nanoparticles could be combined with other treatments for even better results.

## 2 Mechanisms of ferroptosis in cancer cells

### 2.1 Iron metabolism, accumulation and regulation

Iron is involved in several major biological processes. It includes cellular respiration, heme production, enzyme activity, DNA and RNA synthesis, immune function and metabolism (Winter et al., 2014). Normal and cancer cells depend on iron for survival and growth (Lee and Roh, 2023a; Crescenzi et al., 2023). The dysregulation of iron balance can lead to pathological conditions such as tumor initiation, proliferation, and metastasis (Liu J. et al., 2022; Crescenzi et al., 2023). However, excessive production of iron can have adverse effects on cellular components, leading to cell death. Understanding the mechanisms involved in iron uptake, storage, and release within cells is essential for developing effective strategies to target cancer cells (Crescenzi et al., 2023).

Iron can enter the cell through different routes depending on its source (Ma S. et al., 2023). In the ferric form (Fe3+), iron from dietary sources is converted to ferrous iron (Fe2+) by the action of duodenal cytochrome B (DyctB) and then transported into the cell via the divalent metal transporter 1 (DMT1) (Ma X. et al., 2023; Crescenzi et al., 2023). In the bloodstream, Fe3+ is taken up by the cell through the transferrin-transferrin receptor 1 (TF-TFR1) system (Akashi et al., 2021; Qu et al., 2022). Once inside the endosome, Fe3+ is converted to Fe2+ by the six-transmembrane epithelial antigen of the prostate 3 (STEAP3) and then transported into the cytoplasm via endosomal DMT1, contributing to the formation of the labile iron pool (LIP). Excess Fe2+ is exported by the transmembrane exporter ferroportin (FPN) and converted to Fe3+(Yanatori et al., 2010; Velkova et al., 2023).

Other transporters are involved in the transport of iron into the cell cytoplasm or mitochondria. Heme, a circulating form of iron, can enter the cell by endocytosis and then be degraded to Fe2+ by the action of heme oxygenase 1 (HMOX1) (Yanatori et al., 2010; Wang Luping et al., 2023). Multiple divalent metal transporters, such as ZRT/IRT-like protein 14 (ZIP14) or ZIP8, can carry Fe2+ into the cell cytoplasm (Lee and Roh, 2023b). Fe2+ can enter mitochondria through the complex mitoferrin 1/2 (MFRN 1/2)/ATP-binding cassette subfamily B member 10 (ABCB 10) enabling the synthesis of heme and iron-sulfur (Fe-S) clusters. The Fe-S clusters are involved in the generation of reactive oxygen species (ROS), which ultimately contribute to lipid peroxidation and the induction of ferroptosis (Lee and Roh, 2023b).

Regulation of the labile iron pool (LIP) in the cell is tightly controlled by iron transporters involved in iron entry and exit. Iron regulatory proteins (IRPs), notably IRP1 and IRP2, play an important role in this mechanism. Under conditions of iron deficiency, IRPs bind to the 5′untranslated region (UTR) of FPN, reducing iron export and storage. Similarly, IRPs bind to the 3′UTR of the TFR, preventing iron degradation and increasing iron uptake. Conversely, during iron replenishment, the binding of IRP1 to the Fe-S cluster induces a conformational change, inhibiting its binding activity to ferritin or FPN. IRP2, regulated by the iron sensor F-box and leucine-rich repeat protein 5 (FBXL5), undergoes ubiquitination and degradation, preventing its activity (Zhou and Tan, 2017; Cardona and Montgomery, 2023).

In addition to regulating iron transport and storage, cells can also release iron through ferritinophagy. Nuclear receptor coactivator 4 (NCOA4) facilitates the proteolysis of lysosomes, releasing iron from ferritin (Mancias et al., 2014). Nuclear factor erythroid 2-related factor 2 (NRF2), which is controlled by Kelch-like ECH-associated protein 1 (Keap1) also plays an important role in iron metabolism by regulating the activity of NCOA4 and HMOX1 (Fan et al., 2017; Lee and Roh, 2023c).

### 2.2 ROS generation

Reactive oxygen species (ROS), which are part of the reactive species family, are highly reactive oxidants derived from molecular oxygen (O2) (Sies et al., 2022). ROS can be generated enzymatically or nonenzymatically within cells and play an essential role in various cellular functions (Sies et al., 2022). Several studies have demonstrated the involvement of ROS in several cell death processes, including apoptosis, autophagy and ferroptosis (Wang Ling et al., 2023).

Excessive accumulation of Fe2+ in the cell in the form of LIP is one of the mechanisms by which ROS can be generated (Huang J. et al., 2023). When it accumulates excessively, Fe2+ can trigger the Fenton reaction, oxidizing to Fe3+. Electron transfer occurs in the presence of hydrogen peroxide (H2O2), resulting in the formation of the hydroxyl radicals (HO•), a highly reactive type of ROS. (38). The mitochondria and enzymes of the nicotinamide adenine dinucleotide phosphate (NADPH) oxidase (NOX) family also play important roles in ROS generation (Tang and Kroemer, 2020; Zeng K. et al., 2023).

### 2.3 Lipid peroxidation

Lipid peroxidation is a complex process that occurs when reactive oxygen species (ROS), such as HO•, damage unsaturated fatty acids in cell membranes. Lipid peroxidation occurs via two mechanisms: enzymatic and nonenzymatic (Ayala et al., 2014).

Nonenzymatic lipid peroxidation is triggered by the Fenton reaction, where an excess of intracellular Fe2+ reacts with H2O2 to produce HO•. HO• then initiates the oxidation of polyunsaturated fatty acyl chains (PUFAs) by extracting hydrogen from them, forming carbon-centered phospholipid radicals that react with oxygen to form lipid peroxide radicals (PLOO•). These PLOO• radicals propagate the chain reaction by extracting hydrogen from adjacent PUFAs, resulting in the formation of lipid hydroperoxides (PLOOH) and new lipid free radicals. This process triggers further lipid peroxidation. The process is terminated by radical-trapping antioxidants or by reduction catalyzed by glutathione peroxidase. This termination process forms alkoxy lipid radicals (PLO•), which react with PUFAs from another phospholipid (PL), producing PL alcohol (PLOH) and a new PL•. This perpetuates the lipid radical chain reaction (Mortensen et al., 2023).

Several key enzymes, including lipoxygenase (LOX), cytochrome P450 oxidoreductase (POR) and cyclooxygenases (COXs), control enzymatic lipid peroxidation. PUFAs can be synthesized through the action of acyl-CoA synthetase long-chain family member 4 (ACSL4). ACSL4 binds PUFAs to coenzyme A (CoA) to produce PUFA-CoA. Subsequently, lysophosphatidylcholine acyltransferase 3 (LPCAT3) re-esters PUFA-CoA into phospholipids. The most abundant PUFAs in cells, linoleic acid, and arachidonic acid, can be oxidized into PLOOH by the action of LOX, a nonheme iron dioxygenase. LOX catalyzes the dioxygenation of free and esterified PUFAs, generating various PLOOH. In the presence of ferrous iron, PLOOH can breakdown into alkoxy PLO• which further propagates lipid peroxidation or is converted into 4-hydroxynonenal (4-HNE) and malondialdehyde (MDA). These compounds can lead to the formation of protein adducts and disrupt protein structure and function. This in-depth exploration offers valuable insights into the complex mechanisms and regulation of lipid peroxidation, highlighting its critical role as a hallmark of ferroptosis (Bayır et al., 2020; Endale et al., 2023).

### 2.4 Glutamate synthesis, GPX4, and their significance in ferroptosis regulation

Glutamate synthesis and its regulation play a crucial role in the induction of ferroptosis (Yao et al., 2021). This synthesis is dependent on the proper functioning of the system xc-, which comprises the light chain SLC7A11 and the heavy chain SLC3A2. System xc-operates as a cystine/glutamate antiporter and is responsible for importing cystine into the cell and exporting glutamate. Once inside the cell, cystine is reduced to cysteine, which serves as a key element in the synthesis of reduced glutathione. (GSH) (Li B. et al., 2022).

Glutathione peroxidase 4 (GPX4) is a critical regulator of lipid peroxidation, acting to reduce PLOOH to PLOH, thereby preventing the damaging spread of lipid peroxidation (Ursini and Maiorino, 2020; Xu et al., 2021).

Significantly, GSH acts as a vital cofactor for GPX4’s lipid peroxide reductase activity. GPX4 utilizes its catalytic selenocysteine residue and relies on the two electrons donated by GSH, low-molecular thiols, or protein thiols to efficiently counteract lipid peroxidation (Ursini and Maiorino, 2020).

## 3 Ferroptosis resistance mechanisms

A number of advances have been made in the search for a ferroptosis treatments for cancer. Several molecules have been demonstrated to be able to effectively induce cancer cell death via ferroptosis. These include erastin, RSL3, sorafenib, cisplatin and others. Like all therapeutic approaches, ferroptosis induction faces several challenges. Resistance is one such challenge. Several biomarkers whose expression in cancer cells renders ferroptosis-based treatment ineffective have been discovered. The FerrDb database is a free and open resource for identifying ferroptosis-suppressor genes. (http://www.zhounan.org/ferrdb/) (Zhou H. et al., 2023). Several other ferroptosis suppressors have recently been discovered. Table 1 summarizes these ferroptosis suppressors and their mechanisms of action.

**TABLE 1:** Recent ferroptosis suppressors discovered in cancer cells.

Ferroptosis suppressors	Mechanism	Cancer type	References
PDIA4	Upregulate SLC7A11 via PERK/ATF4 pathway	Renal carcinoma	Kang et al. (2023)
SLC12A5	Target the xCT system, Increase GPX4 and decrease ACSL4	Hepatocellular carcinoma	Tong et al. (2023)
CST1	GPX4 upregulation	Gastric cancer	Li et al. (2023a)
FOXA2	GPX4 upregulation	Colorecta cancer	Liu et al. (2023d)
Creatinine kinase B	Phosphorylate GPX4	Hepatocellular carcinoma	Wu et al. (2023)
CEBPG	Transcriptional control of SLC7A11	Ovarian cancer	Zhang et al. (2023b)
CYP1B1	ACLS4 ubiquitination and degradation	Colorectal cancer	Chen et al. (2023b)
TXNCD12	Inhibits lipid peroxidation	-	Tang et al. (2023a)
ACSL1	Upregulate FSP1	Ovarian cancer	Zhang et al. (2023c)
TRIM21	Upregulate FSP1	-	Gong et al. (2023)
P4HA1	Activates HMGCS1	Nasopharyngeal carcinoma	Zhou et al. (2023d)
LOXL3	Prevents DHODH-K344 ubiquitination	Liver cancer	Zhan et al. (2023)
B7H3	Downregulate SREBP2	Colorectal cancer	Jin et al. (2023a)
MBOAT1/2	Phospholipid remodeling	Breast cancer	Liang et al. (2023a)
USP8	Deubiquitinate ß-catenin	Hepatocellular carcinoma	Tang et al. (2023b)
CYBB	Bind to Nrf2, upregulate Nrf2 and SOD2	Glioblastoma	Su et al. (2023b)
Galectin-1	Trigger the phosphorylation of AXL receptor tyrosine kinase (AXL) and MET proto-oncogene, receptor tyrosine kinase (MET) signaling	Hepatocellular carcinoma	Hsu et al. (2023)
Apoc1	Promote GPX4 expression	Esophageal cancer	Hu et al. (2024)
cGAS	Affect mitochondrial ROS accumulation by facilitating dynamin-related protein 1 (DRP1) oligomerization	-	Qiu et al. (2023)
ASS1	Inhibit glutaminolysis, and interfere with TCA	Non-small cell lung cancer	Hu et al. (2023)
CDH4	Promoter GPX4 expression	Oral squamous cell carcinoma	Xie et al. (2023)
SRSF1	Block the ubiquitination of GCH1 and promote it expression	Triple negative breast cancer	Song et al. (2024)
GATA-1s	Prevent for lipid peroxidation	Myeloid leukemia cells	Trombetti et al. (2023)
NSUN2	m5C modification of SLC7A11 and increase in the SLC7A11 level	Endometrial cancer	Chen et al. (2024)
FOXQ1	Promoting GPX4 expression by encouraging miR153 sponging	Breast cancer	Huang et al. (2023e)
TRIM6	Inhibit glutamine import by inducing ubiquitination and degradation of SLC1A5	Lung cancer	Zhang et al. (2023d)
TIMP1	Activation of the PI3K/Akt pathway, promote GPX4 expression	Colorectal cancer	Wang et al. (2023b)
TRIM26	Enhance GPX4 stability	Glioma	Wang et al. (2023c)
ACAT2	Negatively affect ROS generation and lipide peroxidation, promote GPX4 expression	Esophageal squamous cell carcinoma	Heng et al. (2023)
TMEM147	Increase 27HC activity	Hepatocellular carcinoma	Huang et al. (2023a)
Acod1	Mediation of Nrf2-dependent resistance via itaconate, which blocks Keap-1	Breast cancer	Zhao et al. (2023d)
ANO1	Activating PI3K-Akt Signaling	Gastric cancer	Jiang et al. (2023)
CARM1	Accelerating ACSL4 degradation via ACSL4 methylation	Colorectal cancer	Feng et al. (2023b)
CDK1	ACSL4 degradation	Colorectal cancer	Zeng et al. (2023b)
NeuroD1	Promote GPX4 expression	Hepatocellular carcinoma	Huang et al. (2023b)
TEAD1	Upregulate SLC3A2, mTORC1 signal activation	Hepatocellular carcinoma	Li et al. (2023a)
RPLP2	Promote GPX4 expression	Hepatocellular carcinoma	Guo et al. (2023b)
METTL9	Promote SLC7A11 expression	Hepatocellular carcinoma	Bi et al. (2023)
METTL16	Promote GPX4 expression	Breast cancer	Ye et al. (2023)
TSPO	Nrf2 stabilization by P62 accumulation	Hepatocellular carcinoma	Zhang et al. (2023e)
POLE2	Activates Nrf2/GPX4 pathway	Gastric cancer	Jian et al. (2024)

### 3.1 SLC7A11/GPX4

GPX4 expression, as previously stated, inhibits lipid peroxidation. Several studies have revalated proteins that can upregulate GPX4. Most ferroptosis-inducing drugs become ineffective in these situations. Protein disulfide isomerase A member 4 (PDIA4) suppresses ferroptosis via endoplasmic reticulum stress. SLC7A11 is targeted by the latter via the PERK/ATF4 pathway. Salinomycin has recently been shown to suppress PDIA4 expression, thereby increasing ferroptosis (Kang et al., 2023). SLC12A5 is an ion transporter whose expression is increased in hepatocellular carcinoma. It focuses on the xCt system, increasing its activity. It increases GPX4 expression while decreasing ACSL4, resulting in ferroptosis resistance (Tong et al., 2023). The Cystatin SN, encoded by the CST1 gene, has been linked to gastric cancer metastasis. It also suppresses ferroptosis. It was discovered that the CST1 protein and GPX4 have a strong interaction. This interaction stabilizes the GPX4 protein and increases its expression when CST1 levels increase. CST1 relieves GPX4 ubiquitination via the deubiquitinase OTUB1 (Li D. et al., 2023).

Forkhead box transcription factor A2 (FOXA2) is a biomarker capable of increasing GPX4 activity in colorectal cancer (CRC) (Liu J. et al., 2022). Another GPX4 promoter is creatatine kinase B. By phosphorylating GPX4, creatine kinase B can suppress ferroptosis (Wu et al., 2023).

### 3.2 Lipid peroxidation

As lipid peroxidation is the key component of ferroptosis, its inhibition is an obstacle to ferroptosis-based therapy. Several studies have identified biomarkers capable of directly inhibiting lipid peroxidation. TXNDC12 (thioredoxin domain-containing protein 12) is a protein found primarily in the endoplasmic reticulum. When expressed, it inhibits lipid peroxidation independently of the GPX4 pathway. Its upregulation has been shown to be mediated by the expression of the transcription factor ATF4 (Tang J. et al., 2023). ASCL4 plays an important role in lipid peroxidation. When it is inhibited, lipid peroxidation is also inhibited, rendering cancer cells resistant to ferroptosis. Cytochrome P450 1B1 (CYP1B1), promotes ACLS4 ubiquitination and degradation in colorectal cancer (Chen C. et al., 2023).

### 3.3 FSP1/CoQ10 axis and the mevalonate pathway

Ferroptosis suppressor protein 1 (FSP1) is one of the main regulators of ferroptosis. FSP1 converts CoQ10 to CoQ10H2 by consuming NADH/NADPH. CoQ10H2 acts as an antioxidant, preventing lipid peroxidation. FSP1 upregulation is thus a factor in resistance to ferroptosis (Li H. et al., 2023). ACSL1 has been shown to upregulate FSP1 in ovarian cancer (Zhang Q. et al., 2023). FSP1 can convert vitamin K (VK) to VKH2. VKH2 then acts as an antioxidant by scavenging free radicals and thus inhibiting lipid peroxidation (Mishima et al., 2022; Jin D.-Y. et al., 2023). The tripartite motif family member TRIM21 can ubiquitinating the FSP1 protein. Overexpression of TRIM21 promotes FSP1 translocation into the plasma membrane and causes ferroptosis resistance (Gong et al., 2023). CoQ10 synthesis requires the mevalonate pathway (Zheng and Conrad, 2020). Certain enzymes can cause ferroptosis resistance by acting on this pathway. This is the case for P4HA1, a subtype of the prolyl 4-hydroxylase (P4H) family. In fact, P4HA1 can upregulate HMGCS1, a key enzyme in the mevalonate pathway, and thus induce ferroptosis resistance (Zhou N. et al., 2023).

### 3.4 DHOHD/CoQ2 axis

Dihydroorotate dehydrogenase (DHODH) is a mitochondrial enzyme that, like FSP1, inhibits lipid peroxidation (Wang Qin et al., 2023). It catalyzes the conversion of dihydroorotate (DHO) to orotate (OA), as well as the reduction of CoQ to CoQH2. CoQH2 prevents lipid peroxidation. Lysyl oxidase-like 3 (LOXL3) in mitochondria induces ferroptosis resistance by stabilizing DHODH. In effect, LOXL3 prevents DHODH-K344 ubiquitination, which promotes CoQ reduction to CoQH2 in mitochondria (Zhan et al., 2023).

### 3.5 GCH1/BH4 axis

Guanosine 5′-triphosphate cyclohydrolase 1 (GCH1) is an enzyme that regulates ferroptosis. It catalyzes the reaction that produces tetrahydrobiopterin (BH4) from GTP. When GCH1 is overexpressed, BH4 biosynthesis increases, preventing lipid peroxidation. Recently, it was discovered that SR-rich splicing factor 1 (SRSF1) can increase GCH1 expression by preventing its ubiquitination. First, SRSF1 binds to circSEPT9, increasing its expression. In turn, circSEPT9 binds to GCH1, preventing its ubiquitination (Song et al., 2024).

### 3.6 Cholesterol metabolism

Cholesterol biosynthesis is governed by the mevalonate pathway. Isopentenyl pyrophosphate (IPP) is one of the byproducts of cholesterol synthesis. IPP stimulates the production of GPX4 (Zheng and Conrad, 2020). Cholesterol has also been shown to increase ferroptosis resistance via the SLC38A9-mTOR axis by upregulating GPX4. Furthermore, cholesterol accumulation inhibits ferritinophagy in long term hematopoietic stem cells via the SLC38A9-mTOR axis (Liu C. et al., 2023). B7H3 is a ferroptosis inhibitor that interacts with cholesterol biosynthesis. B7H3 downregulates sterol regulatory element binding protein 2 (SREBP2), a transcription factor that plays an important role in regulating cholesterol metabolism by activating the ATK pathway (Jin H. et al., 2023). 7-Hydroxycholesterol (27HC), a common circulating cholesterol metabolite, is a regulator of cholesterol biosynthesis (Liu W. et al., 2021). 27HC increases GPX4 expression and inhibits lipid peroxidation. According to one study, TMEM147 may increase 27HC activity by activating the enzyme 7-dehydrocholesterol reductase (DHCR7) (J. Huang L. et al., 2023).

### 3.7 Sex hormones

Recently, two genes whose expression is controlled by sex hormone were shown to cause ferroptosis resistance. Indeed, two Membrane Bound O-Acyltransferase Domain Containing (MBOAT1/2) genes have been implicated in ferroptosis resistance. Compared with GPX4 and FSP1, MBOAT1 and MBOAT2 induce resistance to ferroptosis via a different. They are controlled by two sex hormone receptors, the estrogen receptor and the androgen receptor. MBOAT1 and MBOAT2 are indeed upregulated by these two receptors. (Liang D. et al., 2023).

### 3.8 Wnt/ß-catenin pathway

The wnt/ß-catenin pathway plays an important role in the regulation of the body’s homeostasis (Liu S. et al., 2022). It has been found that its abnormal activation is involved in the progression of several types of tumors. Several studies have shown that beta-catenin plays a role in cancer cell resistance to ferroptosis. By binding to a transcription factor, ß-catenin can promote GPX4 activity and thus induce resistance to ferroptosis. The transcription factor TCF4, by binding to beta-catenin, induces resistance to ferroptosis. TCF4 has been identified with upregulated expression in gastric cancer. One study showed that it targets GPX4 by upregulating its expression, rendering cancer cells resistant to ferroptosis (Wang Jingmei et al., 2022). Another involves the deubiquitinating enzyme USP8. USP8 has been shown to deubiquitinate ß-catenin by removing the K48-linked ubiquitin chain. By doing so, USP8 stabilizes ß-catenin, resulting in ferroptosis resistance (Tang L. et al., 2023).

### 3.9 Tricarboxylic acid cycle

The mitochondrial tricarboxylic acid (TCA) cycle is necessary for ROS production. The TCA cycle is fed by glutamine metabolism (glutaminolysis). Glutaminolysis via the enzyme glutaminase (GLS2) is therefore necessary for ferroptosis induced by cysteine deprivation (Gao et al., 2019). Clearly, glutaminolysis inhibition is an important factor in ferroptosis resistance. ASS1, a key enzyme in the urea cycle, is important for ferroptosis resistance. ASS1 interferes with the tricarboxylic acid oxidative cycle from glutamine anaplerosis and promotes the reductive carboxylation of cytosolic glutamine, leading to a reduction in ROS production by mitochondria (Hu et al., 2023).

## 4 Nanoparticles for ferroptosis induction in cancer therapy

One of the most effective methods for inducing ferroptosis is the use of nanotherapy. It enables the combination of multiple inducers targeting different pathways. As a result, it is the most effective method for dealing with resistance.

**FIGURE 1:**
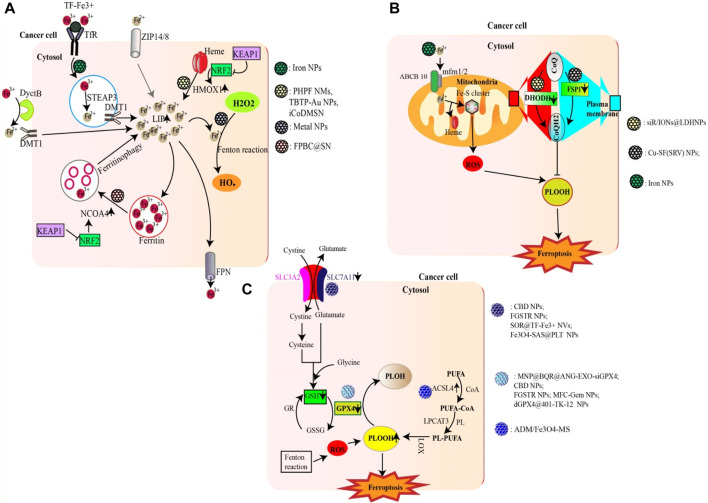
Nanoparticles targeting major metabolic pathways involved in ferroptosis. **(A)** NPs can promote ferroptosis by increasing the LIP (e.g., Iron NPs) either through an Fe2+/Fe3+ ion supply, HMOX1-mediated heme degradation (e.g., FPBC@SN) or ferritinophagy (e.g., PHPF NMs). Excess Fe2+ from LIP as well as metal ions (e.g., Cu2+; Mn2+; Au (I)) generated by gold nanoparticles can trigger the fenton reaction generating excess ROS (HO•). **(B)** Certain NPs are able to inhibit the activity of DHODH (e.g., siR/IONs@LDH) and FSP1 (e.g., Cu-SF(RSV) NPs), thus reducing the production of CoQH2, an inhibitor of lipid peroxidation. The Fe2+ ions supplied by iron NPS can also enter mitochondria and promote ROS production. **(C)** Some NPs can increase lipid peroxidation either by inhibiting the Xc system (e.g., FGSTR NPs) or GPX4 (e.g., CBD NPs). On the other hand, other compounds can increase ACSL4 activity, thereby promoting lipid peroxidation (e.g., ADM/Fe3O4-MS).

Emerging evidence has highlighted the involvement of specific nanoparticles (NPs) in the modulation and initiation of ferroptosis (Acosta et al., 2022; Sun et al., 2022) (Figure 1).

Iron nanoparticles stand out as prime candidates that induce ferroptosis by enhancing the cell’s LIP through the release of iron ions (Zhu G. et al., 2022). In addition to iron nanoparticles, certain NPs have the potential to fast-track ferritinophagy. NPs based on Polyherbal formulations and the combined use of doxorubicin with gold nanoparticles have been shown to accelerate ferritinophagy, leading to the degradation of ferritin and consequently increasing the LIP (Chittineedi et al., 2023). Additionally, certain nanoparticles (e.g., FPBC@SN) have been noted to upregulate NCOA4 activity, revealing yet another mechanism through which nanoparticles affect ferritinophagy (Zuo et al., 2021). In addition to catalyzing ferritinophagy, certain NPs can amplify the activity of HMOX1, as observed with PHPF NMs (Zhang R. et al., 2023). Furthermore, some nanoparticles, such as iCoDMSN, have been shown to modulate the interactions between NRF2 and KEAP1 (Zhao et al., 2023a).

Certain nanoparticles can enhance ferroptosis by inducing substantial amounts of ROS. A prime example includes metal NPs and NPs specifically designed for therapeutic modalities such as phototherapy, radiotherapy, and sonotherapy (e.g., BDPB NPs) (Zheng G. et al., 2023; Chen D. et al., 2023).

The xc-system, which plays a central role in glutathione synthesis, is a possible target for ferroptosis-inducing nanoparticles. By acting on this system (inhibiting SLC7A11), nanoparticles such as SOR@TF-Fe3+ NVs and Fe3O4-SAS@PLT have shown potential for ferroptosis induction (Xiao et al., 2023). In addition, nanoparticles such as MFC-Gem NPs and dGPX4@401-TK-12 can either inhibit the synthesis of GPX4 or reduce GSH, both of which are essential components in the regulation of lipid peroxidation (Luo et al., 2022; Zhang et al., 2022).

The upregulation of lipid peroxidation is crucial to ferroptosis (Krusenstiern et al., 2023). Some nanoparticles, such as ADM/Fe3O4-MS, have been found to increase lipid peroxidation by upregulating ACSL4 (Chen M. et al., 2022). Others, such as siR/IONs@LDH NPs, inhibit DHODH, while Cu-SF(SRV) NPs inhibit FSP1, contributing to the intricate nanoparticle-mediated modulation of lipid peroxidation and ferroptosis (Yang et al., 2022; Chen S. et al., 2023).

## 5 Types of nanoparticles capable of inducing ferroptosis in cancer cells

### 5.1 Metal nanoparticles in cancer therapy

**FIGURE 2:**
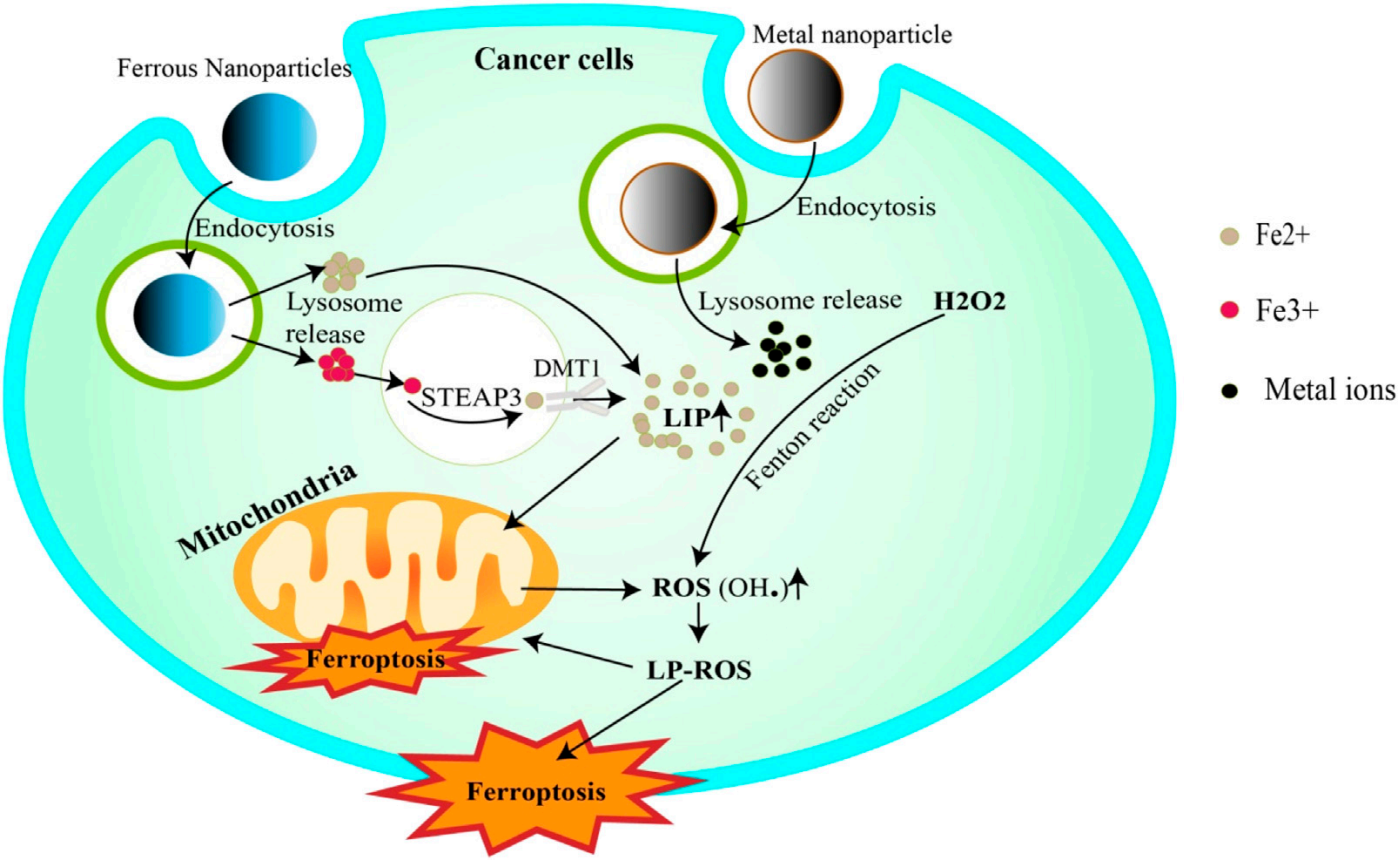
Metal nanoparticles trigger ferroptosis. Iron NPs release Fe3+/Fe2+ ions into the cell via endocytosis. Fe3+ is reduced to Fe2+ by STEAP3 and then completes the LIP. Fe2+, on the other hand, can be added directly to LIP. In addition, LIP releases Fe2+ to trigger the Fenton reaction, or to penetrate mitochondria to generate ROS. ROS attack cell and mitochondrial membrane lipids, inducing their degradation (ferroptosis). Other metallic NPs can also increase ROS generation by releasing ions that trigger Fenton reactivation.

Metal nanoparticles have shown significant promise in cancer therapeutics due to their unique physicochemical properties, particularly their ability to deliver metal ions to cancer cells, stimulating ferroptosis (Dong et al., 2022; Chen X. et al., 2023; Zhong et al., 2023). (Figure 2) This programmed cell death, triggered by metal ion-dependent lipid peroxidation, represents a promising new approach for cancer treatment.

In addition to delivering metal ions, metal nanoparticles can be combined with established ferroptosis-inducing agents including drugs (e.g., sorafenib, dihydroxyartemisinin, RSL3) and noncoding RNAs (miRNAs, siRNA) (Guan et al., 2020; Wang Jianxin et al., 2022; Guo et al., 2022; Huang et al., 2022).

#### 5.1.1 Ferrous nanoparticles

Ferrous nanoparticles designed to deliver ferrous ions have been identified as effective agents for inducing ferroptosis in cancer cells. The delivered ions generate reactive oxygen species (ROS) through the Fenton reaction, leading to lipid peroxidation and, subsequently, ferroptosis (Chi et al., 2022).

Iron oxide nanoparticles (IONs), a subtype of ferrous nanoparticles, have gained increased research attention due to their ability to induce ferroptosis across various types of cancers both *in vitro* and *in vivo* (Chen Z. et al., 2022; Chen Y. et al., 2022; Chin et al., 2022). One specific example includes a type of ION synthesized by Huang et al., which successfully induced ferroptosis in diffuse large-cell B lymphoma by introducing iron ions into target cells. This promoted the generation of ROS through the Fenton reaction, leading to lipid peroxidation and cell membrane destruction. Additionally, these IONs influence the regulation of iron metabolism-related proteins, such as ferroportin (FPN) and transferrin receptor (TFR), thus increasing the intracellular labile iron pool (LIP) (Q.-T. Huang P. et al., 2023).

Moreover, IONs can serve as a core for creating multifaceted nanoparticles that combine various components (biopolymers, ferroptosis-inducing agents, peptides), enhancing their therapeutic effect (Lin et al., 2022; Liang Z. et al., 2023). For example, the Fe3O4-PEI@HA-RSL3 nanoparticle developed by Liang et al., has demonstrated great potential for inducing ferroptosis in hepatocellular Carcinoma (HCC) by enhancing ROS production and suppressing several ferroptosis-related genes (Han et al., 2022). Another complex nanoparticle is MNP@BQR@ANG-EXO-siGPX4 which was designed by Boyan Li et al., can target glioblastoma cells via its CD36 antibody and effectively release Fe2+ ions (Li F.-J. et al., 2022).

Another strategy involves the integration of iron ions (Fe2+ or Fe3+) into the nanoparticle system through chelation. These ions can be derived from FeCl2 or FeCl3 and can be added to other nanoparticles through chelation (UPDA-PEG@Fe2+/3+) (Chen et al., 2019a). Fe (acac)3 and FeSO4 can also serve as sources of Fe3+ and Fe2+ respectively when incorporated into nanoparticles (Zhang S. et al., 2023; Zhong et al., 2023).

The use of ferrocene, a biocompatible, hydrophobic, low-toxicity, and redox-friendly organometallic compound, has been effective in designing ferroptosis inducing nanoparticles (Wang Yingjie et al., 2022). For instance, Lin et al. created a nanoparticle (mPEG-b-PPLGFc@Dox) by assembling ferrocene with methoxypolyethylene glycol-b-poly (γ-3-azidopropanyl-L-glutamate) (mPEGb-PAPLG) and doxorubicin, achieving therapeutic effectiveness in prostate cancer while limiting collateral damage to nontarget cells (Lin et al., 2023).

Moreover, the inclusion of hemin, a potent source of Fe2+ and enhancer of HMOX1 activity, is another strategy for designing ferroptosis-inducing nanoparticles (Su I.-C. et al., 2023). Hemin can also consume glutathione (GSH), inhibiting the activity of GPX4, thereby promoting lipid peroxidation (Xiao K. et al., 2022). Zhang et al. exploited the synergistic potential of hemin and ferrocene to design a PHPF nanomotor, achieving significant antitumor therapeutic effects via ferroptosis (Zhang X. et al., 2023).

#### 5.1.2 Nonferrous nanoparticles

##### 5.1.2.1 Copper nanoparticles

The ability of Cu2+ ions to catalyze the Fenton reaction and produce reactive oxygen species (ROS) has sparked interest in developing copper-based nanoparticles for ferroptosis induction. Several innovative copper-based nanosystems have been designed that capitalize on the redox transformation between Cu2+ and Cu + to generate cytotoxic hydroxyl radicals (OH•) that promote ferroptosis (Yuan et al., 2022).

For instance, the PAA-Cu3(PO4)2-DOX-CMCS nanoparticle, as designed by Sheng Zhao et al., efficiently delivers doxorubicin and Cu2+ ions into 4T1 cells. The released Cu2+ ions undergo a reduction to Cu + by GSH, promoting the Fenton reaction and the subsequent generation of ROS (Zhao et al., 2022). Similarly, the Cu-SF(RSV) NPs, developed by Jie Yang et al., leverage copper ions and silk fibroin (SF) to trigger substantial ROS production, which in tandem with the action of rosuvastatin (RSV), induces lipid peroxidation and eventual ferroptosis (Yang et al., 2022).

##### 5.1.2.2 Nickel nanoparticles

Nickel-based nanoparticles are also under scrutiny for their potential to generate ROS and induce ferroptosis (Yang Qunfang et al., 2023; Wang Weikai et al., 2023). The NiB@IrOx nanoparticles developed by Qin Wang et al. represent an innovation in this field. This nanoparticle effectively induces ROS production and subsequent ferroptosis through the catalytic action of IrOx and nickel (Ni2+/Ni0) in Hepa1-6 tumors in female BALB/c nude mice (Wang et al., 2023g).

##### 5.1.2.3 Cobalt nanoparticles

Cobalt-based nanoparticles, due to their capacity to generate ROS, have shown potential in cancer therapy (Alarifi et al., 2013; Huang et al., 2021). iCoDMSNs, nanoparticles designed by Jianqi Zhao et al., combine cobalt oxide nanodots with iRGD peptides and dendritic mesoporous silica nanoparticles. This complex of nanoparticles selectively accumulates in tumor sites, triggering ferroptosis by inducing ROS production and regulating the expression of KEAP1, NRF2, and HMOX1, which in turn elevates the labile iron pool in 4T1 tumor-bearing BALB/c model mice (Zhao et al., 2023b).

##### 5.1.2.4 Manganese nanoparticles

Manganese ions (Mn2+) can induce ferroptosis in cancer cells by catalyzing the Fenton reaction and generating ROS (Dong et al., 2022). In the quest for novel cancer therapeutics, researchers have utilized this property to develop manganese-based nanoparticles such as PEG-PDA@Mn NPs, which have shown efficacy in gastric cancer therapy (Chen and Wen, 2022).

##### 5.1.2.5 Zinc nanoparticles

Zinc’s role in ferroptosis has been demonstrated in various studies, with zinc oxide nanoparticles (ZONPs) showing potential in cervical and breast cancer treatment (Chen J. et al., 2021; Li Q. et al., 2023). In cervical cancer, ZONPs not only induce lipid ROS and malondialdehyde (MDA) production but also suppress CD164 by enhancing miR-505-3p levels (Lei et al., 2022). In breast cancer, ZONPs have been shown to counteract resistance to doxorubicin by inhibiting ABCC9 and inducing ferroptosis (Li Y. et al., 2022).

##### 5.1.2.6 Gold nanoparticles

The unique ability of gold to induce ferroptosis is being leveraged in the development of gold-based anticancer drugs (Zou et al., 2015; Xiao X. et al., 2022). The released Au(I) ions can inhibit the activity of thioredoxin reductase (TrxR), leading to an increase in ROS levels, rapid lipid peroxidation, and the subsequent induction of ferroptosis (Xiao K. et al., 2022). A notable example is the Au(I)-based NIR-II ferroptosis nanoparticles (TBTP-Au NPs) created by Jing Zhang et al. for glioblastoma treatment. These nanoparticles exploit the high ROS gradient in the glioma microenvironment to trigger the selective binding of Au(I) to overexpressed TrxR, activating HMOX1-regulated noncanonical ferroptosis in glioma cells and leading to tumor suppression (Zhang Xiaoqian et al., 2023).

##### 5.1.2.7 Cerium oxide nanoparticles

Cerium oxide nanoparticles, also known as CeO2 NPs, are another type of NP that can induce ferroptosis (Wang et al., 2022d, p. 53). These nanoparticles work by triggering the Fenton reaction (Gao et al., 2023). Researchers like Xuan Gao and his team have shown that when these CeO2 NPs enter a cell, they can transform H2O2 into HO•. The nanoparticles release ions called Ce(III) when they enter the cell. These ions interact with H2O2 present in the cell to create another form of cerium ion, Ce(IV) and HO•. HO• then induces lipid peroxidation, ultimately leading to cell death through ferroptosis (Gao et al., 2023).

##### 5.1.2.8 Silver nanoparticles

Silver nanoparticles are another type of metal NP that can trigger ferroptosis. These NPs achieve this by ROS (G. Zheng S. et al., 2023). Christina M. Snyder and her team have shown that silver nanoparticles can be particularly effective against triple-negative breast cancer. They do this by causing a lipid peroxidation (Snyder et al., 2021).

### 5.2 Metal-free nanoparticles

Although the use of metal-based nanoparticles in ferroptosis has been explored extensively, the emergence of metal-free nanoplatforms has opened new avenues in cancer therapeutics. Given the high iron content in cancer cells, ferroptosis induction using iron-based materials might lead to iron intoxication after the cancer cells burst and release their contents. Therefore, the development of nonferric, or even better, nonmetallic nanoparticles, provides an alternative approach to induce ferroptosis without this risk.

Cannabinoids (CBDs), have long been implicated in various cell death mechanisms critical to cancer therapy (Salazar et al., 2009; Erzurumlu et al., 2023; Whynot et al., 2023). Recently, studies by researchers such as Qunfang Yang et al. have revealed its potential in stimulating ferroptosis. They found that CBD not only was capable of inducing ROS production but also exhibited regulatory effects on GPX4, SLC7A11 and FTH1 by downregulating them and upregulating ACSL4. Researchers have proceeded to design a nanoparticle named CP NPs, which couples CBD to the Toll-like receptor three agonist, poly (I:C). This ingenious combination facilitated the delivery of CBD into murine melanoma cells, generating a ferroptosis response alongside an enhanced immune reaction induced by poly (I:C) (Yang Qing et al., 2023).

In addition to these strategies, ferroptosis-inducing metal-free nanoparticles can be fashioned by integrating ferroptosis drugs or molecules into nanoparticles. For instance, GPX4 inhibitors such as FIN56 or even GPX4 degraders have been used to develop such nanoparticles (Luo et al., 2022; Sun et al., 2023). Expanding on this concept, Xinxin Sun et al. created a nanoparticle by amalgamating FIN56 with arachidonic acid (AA). A small amount of lipid-PEG containing a disulfide bond (DSPE-SS-PEG2K) was then added to this pair, resulting in redox-sensitive lipid peroxidation nanoamplifiers (FAS NPs). This 'drug-deliver-drug’ nanoparticle strategy leveraged the inherent tendency of AA to augment lipid peroxidation, thereby boosting the ferroptotic response (Sun et al., 2023).

Another intriguing development in this field revolves around a pentadecapeptide derivative of cleaved high molecular weight kininogen (HKa), HKN15. Because of its strong binding affinity for ferritin, HKN15 has been used by Luwen Zhu et al. to create a modified nanoparticle, Ce6-PEG-HKN15 NPs. Upon HKN15-mediated uptake, these nanoparticles were found to congregate around ferritin, triggering its destruction and subsequent release of iron into the cytoplasm. This liberated iron, in turn, catalyzed the production of ROS through the Fenton reaction, successfully driving the cells toward ferroptosis (Zhu L. et al., 2022).

## 6 Overcoming ferroptosis resistance combined conventional therapies and nanoparticles

Traditional therapies such as phototherapy, sonotherapy, microwave-assisted thermal therapy, and radiotherapy have been found to potentiate ferroptosis by generating ROS. Nanoparticles can be engineered to carry agents receptive to each therapy type and can be activated by corresponding stimuli including light, ultrasound, microwaves, or ionizing radiation (Lang et al., 2019; Liu X. et al., 2021; Feng S. et al., 2023; Liu S. et al., 2023).

In the domain of phototherapy, light radiation (spanning near-infrared (NIR), X-ray, or UV light) is used to deliver tumor-inhibiting or tumor-destroying agents. NIR-based therapy is especially beneficial for localized cancers due to its selectivity, low-toxicity, resistance-limiting properties and minimal collateral damage. Moreover, NIR can amplify the ferroptosis response by boosting the Fenton reaction, a process that produces harmful hydroxyl radicals from iron and hydrogen peroxide (Chen Z. et al., 2022; Yu et al., 2022; Zhang et al., 2023h). Researchers, such as Lu Chen et al. and Hongli Yu et al. have developed PDA-based nanoparticles that can chelate with ferroptosis-inducing agents such as iron ions (Chen et al., 2019b; Yu et al., 2023). Particularly notable is the work of Hongli Yu et al. where Fe3+ ion-chelated PDA nanoparticles, covered with a red blood cell membrane, showed significant tumor inhibition under NIR irradiation, which was linked to increased ROS production and reduced GPX4 expression (Yu et al., 2023).

Microwave-assisted thermal therapy, which utilizes microwaves to induce hyperthermia and damage cancer cells, has also been explored in the context of ferroptosis (Chen Y. et al., 2022). Hui Zhou et al.'s work with CuCy nanoparticles exemplifies this approach, demonstrating increased ROS generation and lipid peroxidation, coupled with a reduction in GPX4 expression when subjected to microwaves (Zhou et al., 2023c).

Radiotherapy, a cornerstone of cancer treatment, not only fractures the DNA double bonds but also generates ROS through ionizing radiation (Gong et al., 2021). This ROS-generating property can be harnessed to enhance ferroptosis using nanoparticles sensitive to radiotherapy. For instance, Shuting Zheng et al.'s study presented a nanoparticle design, (HMON)-GOx@MnO2 NPs, which exhibited a pronounced ferroptosis effect following exposure to ionizing radiation (Zheng G. et al., 2023).

Sonodynamic therapy (SDT), similar to radiotherapy, promotes ROS production and can thereby boost the effect of ferroptosis when combined with a ferroptosis inducer (Pan et al., 2020; Zhang et al., 2023i). Recently Jianxin Wang et al. developed nanoparticles (IRP NPs) that amalgamated the sonosensitizer IR780 and a ferroptosis inducer RSL-3, enveloped in biocompatible poly-(lactic-co-glycolic acid) (PLGA). These nanoparticles, which leverage the mitochondrial targeting capabilities of RSL-3, successfully induced ferroptosis by impairing the mitochondria of TNBC cells (Wang Yue et al., 2022).

## 7 The interplay of ferroptosis and immunotherapy in cancer treatment with using nanoparticles

The innovative domain of immunotherapy has joined forces with conventional cancer treatments to create a potent therapeutic approach (J. Chen P.-H. et al., 2021; Formenti et al., 2018; Twyman-Saint Victor et al., 2015). Importantly, the ferroptosis mechanism has been found to enhance these immunological responses, specifically by inducing immune cell death (ICD) (Zhou et al., 2023d).

It begins with the aftermath of ferroptosis: the deceased cancer cells release damage-associated molecular patterns (DAMPs), effectively initiating the immune response. Research has revealed that ferroptosis cells expel DAMPs, specifically high mobility group box 1 (HMGB1), into the tumor microenvironment. These DAMPs, in turn, promote dendritic cell (DC) maturation and lead to the recruitment of a broader range of immune cells, thereby amplifying ICD (Hsieh et al., 2021; Zhou H. et al., 2023).

**FIGURE 3:**
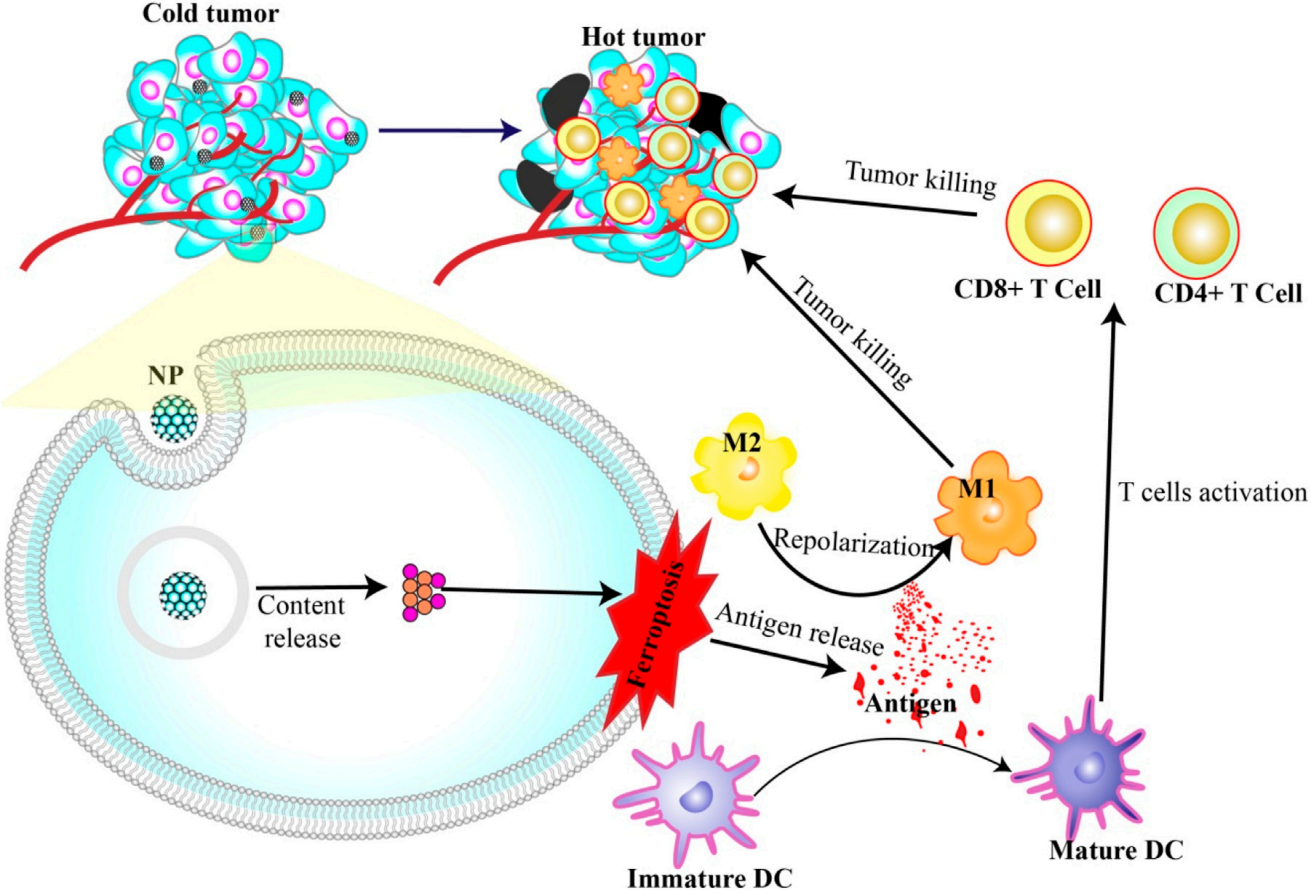
Nanoparticles induce ferroptosis and immunotherapy. Antigens released during NP-induced ferroptosis can activate DC maturation and repolarization of M2 to M1. Mature DCs in turn activate T cells to attack and kill cancer cells.

In another application, nanoparticles capable of inducing ferroptosis have emerged as potential immunostimulants. These nanoparticles help mature dendritic cells through antigens produced during cell death. Once they mature, these dendritic cells recruit T cells to enter the tumor microenvironment and kill more cancer cells. Additionally, the release of DAMPs (e.g., HMGB1 and KRAS) can repolarize M2 macrophages, which usually help tumors grow into M1 macrophages, which actively fight against the tumors (Dai et al., 2020b; 2020a; Jiang et al., 2020; Lei et al., 2023) (Figure 3).

This was aptly demonstrated in a study by the research group, Hsieh et al., 2021. They used zero-valent iron nanoparticles (ZVI-NPs) to stimulate ferroptosis in cancer cells, which not only initiated cell death but also spurred a significant immune response. ZVI-NPs transformed protumor M2 macrophages into antitumor M1 macrophages, reduced the population of regulatory T cells, and downregulated PD-1 and CTLA4 in CD8^+^ T cells to boost their cancer cell cytolytic activity. Notably, they also managed to curtail PD-L1 expression in cancer cells (Hsieh et al., 2021).

Similarly, in 2023, Zhou et al., in 2023, worked with FeGd-HN@Sorafenib@TGF-β-antibody@RGD2 NPs (FG-STR NPs) to treat MC38 tumor-bearing C57BL/6 mice. Treatment results in the infiltration of various immune cells, including memory B cells, eosinophils, lymphoid cells, mast cells, NK cells, monocytes, M1 macrophages, and CD8^+^ T cells, into the tumor microenvironment. This led to the production of IFN-γ+, provoking antitumor cytotoxicity. Moreover, increased expression of markers such as CD11c+, CD80^+^, and MHC-II + confirmed the maturation of DCs (Zhou et al., 2023c).

Building on these findings, specific components can be integrated into the nanosystems to further augment immune cell activation. A key example includes the indoleamine-2,3-dioxygenase (IDO) inhibitors (e.g., NLG919), which are used in the creation of FPBC@SN nanoparticles, as detailed in the work of Zuo et al. This is particularly relevant because an increase in IDO expression can increase tryptophan degradation, which is crucial for T cell activation and proliferation (Zuo et al., 2021).

Another intricate nanoformulation is CP nanoparticles, which are composed of CBD nanoparticles and poly (I:C). As illustrated by Kim et al., these nanoparticles not only inhibit the increase in PD-L1 expression on tumor cells but also enhance tumor sensitivity to T-cell-mediated destruction. This prevents tumor escape from immunosurveillance, thereby improving the overall efficacy of immunotherapy (Yang Qunfang et al., 2023).

## 8 Nanoparticles that induce ferroptosis and other types of cell death (apoptosis, autophagy and cuproptosis)

### 8.1 Nanoparticles as dual agents for inducing ferroptosis and apoptosis

The potential of nanoparticles to promote ferroptosis, has led to promising developments in cancer therapeutics. However, these findings have shed light on an even more intriguing capability: their role in concurrently inducing apoptosis in cancer cells. This dual function serves as a multifaceted attack on cancer cells, opening up the possibility of exploiting multiple cell death pathways for a more comprehensive therapeutic strategy (Zhong et al., 2023).

The common denominator in both ferroptosis and apoptosis induction is the generation of reactive oxygen species (ROS). NPs prompt ROS production, leading to lipid peroxidation, a critical factor in initiating both ferroptosis and apoptosis. The products of lipid peroxidation interact with membrane receptors, transcription factors, or inhibitors, triggering apoptosis signaling. In parallel, ROS can breach the mitochondrial membrane integrity, releasing cytochrome c into the cytoplasm, an event that initiates caspase three activation and thus initiates apoptosis (Su et al., 2019; Wang Yaxuan et al., 2023).

This interplay between ferroptosis and apoptosis induced by nanoparticles has been highlighted in numerous studies. For instance, Yuan et al.'s work with CuCP Lipo nanoparticles demonstrated their dual ability to induce ferroptosis and apoptosis in cancer cells. The nanoparticles initiated lipid peroxidation and triggered the upregulation of the p53 protein, a well-known regulator of cell cycle arrest, senescence, apoptosis, and ferroptosis (Yuan et al., 2022).

A similar pattern was observed in the research conducted by Zhou et al. where HMPB/ML210@TA-BLM-Fe3+ nanoparticles were used to treat breast cancer in a mouse model. Their results reveal elevated caspase three expression in the treated cancer cells, indicating the nanoparticles tended to induce apoptosis and ferroptosis (Zhou et al., 2021).

Another noteworthy aspect of using nanoparticles in cancer therapy is their ability to carry other therapeutic agents, thereby amplifying their overall impact. This was exemplified by the research led by Jiaxin Zhu et al. who developed a nonmetallic nanoparticle known as an ASP NPs. In combination with aurantiamide acetate, scutebarbatine A, and palmitin, these nanoparticles not only induced ferroptosis but also induced apoptosis in breast cancer cells. They increase the expression of apoptosis-related proteins, including cytochrome C, caspase 3, caspase-9, and Bax, while reducing the expression of the antiapoptotic protein, Bcl-2 (Zhu et al., 2023).

### 8.2 Nanoparticle-induced autophagy and ferroptosis: dual cell death pathways in cancer therapeutics

Autophagy-dependent cell death is a crucial regulatory mechanism essential for maintaining cellular equilibrium. It is initiated by a myriad of triggers, including peptide accumulation, endoplasmic reticulum (ER) stress, and nutrient deprivation (Shen et al., 2023). Core to the autophagy machinery is signaling cascades involving the mammalian target of rapamycin (mTOR) and the mitogen-activated protein kinase (AMPK). These factors, in turn, modulate key autophagy-related proteins such as the Unc-51 like autophagy activating kinase 1 (ULK1) complex (Inoki et al., 2012; Wang et al., 2023i).

Interestingly, lipid peroxidation, a marker of cellular oxidative stress, can induce ER stress, subsequently driving autophagy through the PKR-like ER kinase (PERK) pathway. This often leads to the activation of c-Jun-N-terminal kinase (JNK). Integral to this cascade are processes such as the lipidation of microtubule-associated light chain-3 (LC3-I) to form LC3-II and the liberation of Beclin one from its inhibitory association with BCL2, both of which are hallmarks of autophagosome formation and autophagic progression (Wang Zuo et al., 2023).

Reactive oxygen species (ROS), omnipresent in cellular processes, have their own intricate link to autophagy. They can stimulate the adenosine 5′-monophosphate (AMP)-activated protein kinase (AMPK), driving autophagy either through mTOR inhibition or by influencing the ULK complex to release Beclin one from BCL2 (Egan et al., 2015; Zhang Z. et al., 2023; Wang Zhangjie et al., 2023).

Within the realm of cancer research, certain nanoparticles have shown their ability to induce both autophagy and ferroptosis. A prime example is ultrasmall iron oxide nanoparticles (USIONPs). When applied to human glioblastoma cells, these nanoparticles exhibited an increased expression the of autophagic markers Beclin 1/ATG5, alongside markers indicative of ferroptosis (Ren et al., 2019; Wen et al., 2021). Similarly, IONP@PTX NPs mirrored the autophagy and apoptosis induced by USIONPs, as indicated by increases in Beclin 1, LC3 II, LC3 I levels and a concurrent decrease in P62 (Chen and Wen, 2022). Further confirming the dual-role of these nanoparticles, the introduction of Fe2O3@Co-PEG to fibrosarcoma cells, led to autophagy-dependent ferroptosis, as reported by Thanpisit Lomphithak et al. (Lomphithak et al., 2023).

### 8.3 Ferroptosis and cuproptosis: copper nanoparticles as key players

The association of copper with cell death can be traced back to the 1980s. Its involvement was initially thought to be intertwined with reactive oxygen species (ROS) mechanisms, drawing it closer to well-charted cell death processes such as apoptosis, ferroptosis, autophagy, and necroptosis (Halliwell and Gutteridge, 1984). This framework, however, was challenged and redefined in 2022 by Tsvetkov et al. who shed light on “cuproptosis”. In contrast to previous assumptions, this pathway deviates from ROS and focuses on the tricarboxylic acid (TCA) cycle in mitochondria (Tsvetkov et al., 2022).

Lipoylation, a highly conserved posttranslational modification of lysine, has been observed in a select group of enzymes: pyruvate dehydrogenase (PDH), alpha-ketoglutarate dehydrogenase (KDH), branched-chain alpha-ketoacid dehydrogenase (BCKDH), and the glycine cleavage system (GCV). When copper binds to these lipoylated enzymes, particularly within the TCA cycle, it leads to their aggregation, culminating in proteotoxic stress and subsequent cell death (Tsvetkov et al., 2022).

Essential participants in this process include ferrodoxin-1 (FDX1) (a reductase that reduces Cu2+ to Cu+), the lipoic acid enzymes such as lipolytransferase 1 (LIPT1), lipoyl synthase (LIAS), and dihydrolipoamide dehydrogenase (DLD), and key components of the PDH complex and complex I genes. The PDH complex includes pyruvate dehydrogenase E1 subunit alpha 1 (PDHA1), pyruvate dehydrogenase E1 subunit beta (PDHB), and dihydrolipoamide S-acetyltransferase (DLAT) (Wang Zhenjie et al., 2022; Tsvetkov et al., 2022).

A therapeutic breakthrough has emerged in the form of the chemotherapy agent, Elesclomol (ES). When paired with copper, ES has emerged as a promising initiator of curroptosis, signifying its ability to target mitochondria and induce this specialized form of cell death (Hasinoff et al., 2014; Li Yongyun et al., 2022; Liu T. et al., 2023).

In addition to inducing ferroptosis, copper nanoparticles (Cu NPs), play a role in inducing cuproptosis, making them particularly useful for treating cancer. For example, a study by Boda Guo’s team developed a specific copper nanoparticle called NP@ESCu that successfully induced cuproptosis in mice with bladder cancer. These nanoparticles successfully targeted mitochondria using ES. NP@ESCu not only delivers copper ions into the cells but also generates reactive oxygen species (ROS) and initiates an immune response (Guo B. et al., 2023).

When Cu NPs penetrate cells, they release copper ions (Cu2+; Cu+). Cu2+ then interacts with GSH, leading to the increased production of ROS. This influx of ROS helps induce another forms of cell death (apoptosis, ferroptosis and autophagy) and contributes to the rapid accumulation of Cu+ in the cell (Halliwell and Gutteridge, 1984; Zhao et al., 2023a). This excess Cu + can bind to a protein named DLAT, causing it to clump together. This clumping, also known as agglomeration, creates a type of harmful stress in the cell that activates cuproptosis. Additionally, the copper ions are capable of inhibiting proteins that contain iron-sulfur groups, which also leads to harmful stress in the cell and triggers cuproptosis (Zhao et al., 2023b).

One research direction involves increasing the level of copper inside cells by blocking GSH (Tsvetkov et al., 2022). This technique can be particularly effective when used alongside copper nanoparticles (Wang Bingqing et al., 2023). Cu-GA NPs, which are copper nanoparticles combined with a chemotherapy drug called galic acid, have shown promise in triggering cuproptosis, while also increasing the production of ROS and inhibiting GSH (Zhao J. et al., 2023).

Furthermore, modulating the copper transporter ATP7B offers a method to optimize intracellular copper concentrations, potentially supercharging therapeutic interventions against cancer (Polishchuk et al., 2014). Fan Zhao et al. have found a way to improve the effectiveness of cuproptosis by inhibiting the copper-transporting protein, ATP7B. They created a unique copper nanoparticle, Cu2(PO4) (OH) NPs, that block the activity of ATP7B, thereby optimizing the intracellular copper concentration and increasing the efficiency of the cuproptosis process (Zhao Y. et al., 2023).

Table 2 lists some recently developed ferroptosis-inducing nanoparticles for combination therapy.

**TABLE 2:** Ferroptosis-inducing nanoparticles in combination therapy.

Nanoparticles	Combination therapy	Mechanism	Cells type	References
Fe-MOF-RP	Ferroptosis, apoptosis, immunotherapy	ROS generation, DCs maturation, Macrophage polarization	B16F10 cells	Fan et al. (2017)
FECTPN	Ferroptosis, apoptosis	ROS generation	MCF-7 cells	Huang et al. (2023d)
FGTL	Ferroptosis, immunotherapy	ROS generation, DCs maturation, macrophages polarization	LLC cells	Zhang et al. (2023a)
ICG@PEG-CS/PDA	Ferroptosis, apoptosis	GSH depletion, ROS generation	CT26 cells	(Chen et al., 2024)
PPAH	Ferroptosis, apoptosis	ROS generation	MCF-7, BT474, MDA-MB231 cells	Zhao et al. (2023c)
OCH	Ferroptosis, apoptosis	GSH depletion, ROS generation	4T1 cells	Shi et al. (2024)
Ce6@Cu	Ferroptosis, Cuproptosis	ROS generation, GSH depletion, DLAT aggregation	U87MG cells	Zhu et al. (2024)
TTHM	Ferroptosis, immunotherapy	Suppression of system Xc−, DCs maturation, CD8^+^ T cell attraction, IFN-γ liberation	4T1 cells	Chen et al. (2024)
CuP/Er	Ferroptosis, cuproptosis, immunotherapy	ROS generation, GSH depletion, Glycolyis decreasing, DLAT lipoylation, DCs maturation and antigen presentation	4T1, MC38 cells	Li et al. (2023d)

## 9 Conclusion and perspective

**FIGURE 4:**
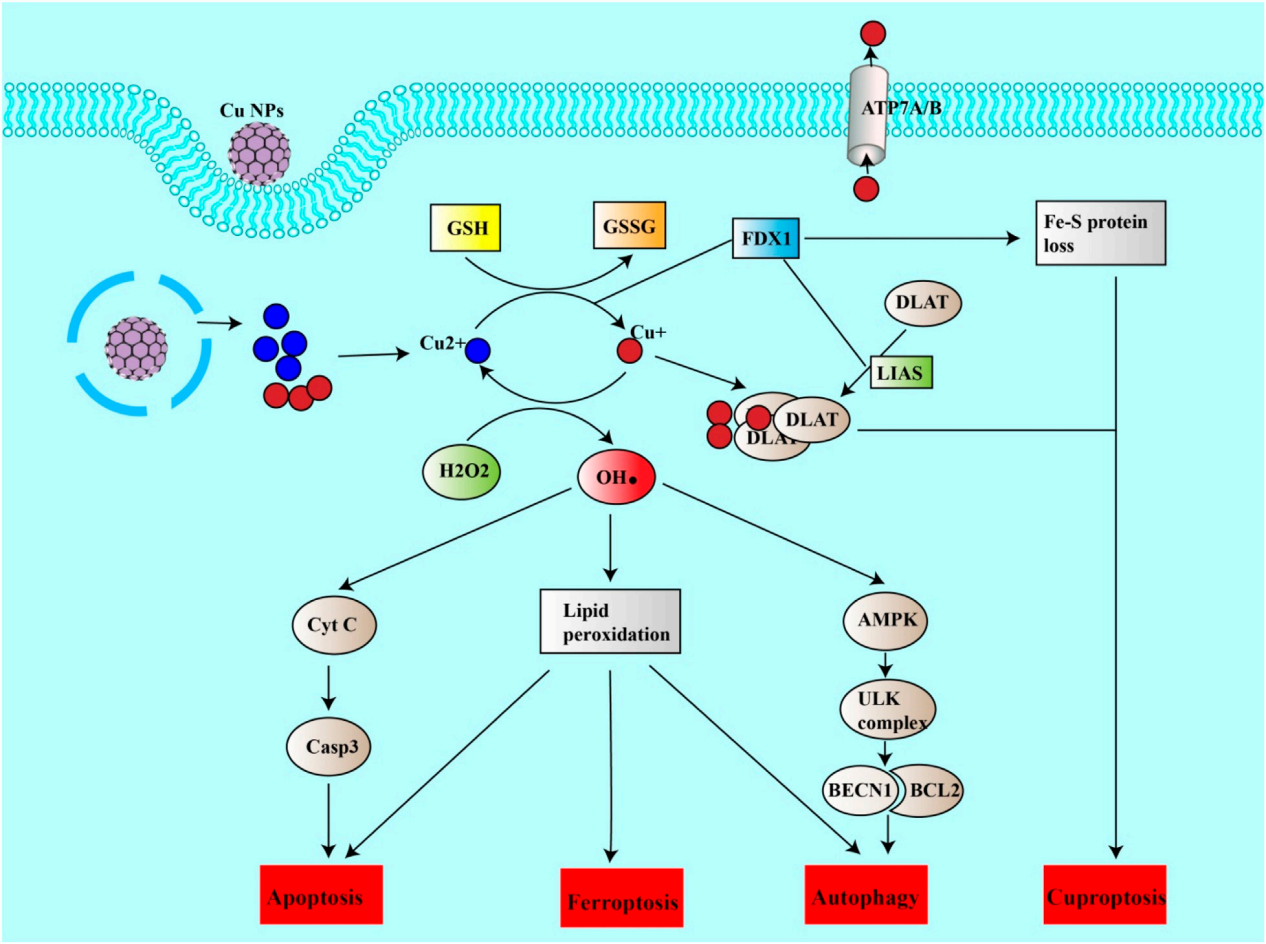
Copper nanoparticles induce cuproptosis and other forms of cell death. Cu NPs can release copper ions into the cell. Cu + can be oxidized to Cu2+, generating ROS (HO). ROS can thus induce lipid peroxidation leading to ferroptosis, apoptosis or autophagy. ROS can itself induce apoptosis by inducing the release of CytC from mitochondria, which in turn activates the casp3 pathway. ROS can also induce autophagy via the AMPK/ULK complex pathway, leading to the release of BECN1. On the other hand, Cu2+ can be reduced to Cu + by the action of FDX1 or by reducing GSH to GSSG. Cu + can thus enter mitochondria, bind to DALT and form a DALT-Cu aggregate under the action of FDX1 and LIAS. This aggregation and the inhibition of sulfide-containing proteins (loss of the Fe-S protein) are the causes of cuproptosis.

Ferroptosis, a form of iron-dependent cell death, has been increasingly recognized for its transformative potential in the landscape of cancer treatment. With distinct mechanisms that differ from those of regular cell death pathways, ferroptosis presents a compelling avenue for circumventing resistance to existing therapies. Recent advancements in nanoparticle-based approaches have further catalyzed this promise, demonstrating multifaceted capabilities, ranging from inducing reactive oxygen species (ROS) to activating other cellular death pathways (Figure 4).

The synergistic benefits of ferroptosis-inducing nanoparticles are particularly salient. These nanoparticles not only cause cell death but they also stimulate an immune response. The DAMPs generated by cell death results in the maturation of dendritic cells (DCs), the recruitment of T lymphocytes and the polarization of M2 macrophages into M1 macrophages. This immunogenicity has proven invaluable in the treatment of cancer in animal models by combining ferroptosis and vaccines (Ma S. et al., 2023; Fang et al., 2023; Shi et al., 2023). Further research is required before these formulations can be tested on humans. The ferroptosis and nanovaccine combination tested today has always been a single nanoparticle formulation. Combining two nanoparticle formulations, one for the ferroptosis and one for the vaccination, could improve the efficacy of the combinatorial effect.

Despite the advantages of ferroptosis-inducing nanoparticles, it is crucial to acknowledge the potential hazards associated with the use of metal nanoparticles and the excessive ROS generation (Makhdoumi et al., 2020). These include toxicity to nearby normal cells and unknown long-term health effects, warranting rigorous *in vivo* evaluations for safety and efficacy. There is an imperative need to develop nanoparticles that are highly selective for cancer cells while sparing normal tissues, in order to minimize unintended consequences. Manufacturing nanoparticles that can target cancer cells exclusively or primarily through their specific receptors may reduce the toxicity to nearby normal cells. The therapeutic combinations used to induce ferroptosis must also be thoroughly researched. The type of cancer as well as the resistance mechanisms associated with that type of cancer must be considered.
